# Role of point-of-care ultrasonography (POCUS) in the diagnosing of acute medial meniscus injury of knee joint

**DOI:** 10.1186/s13089-021-00256-0

**Published:** 2022-02-08

**Authors:** Omid Ahmadi, Mehdi Motififard, Farhad Heydari, Keihan Golshani, Azita Azimi Meibody, Saeed Hatami

**Affiliations:** 1 Department of Emergency Medicine, Faculty of Medicine, Alzahra Hospital, Isfahan University of Medical Sciences, Sofeh Ave, Keshvari Blvd., Isfahan, Iran; 2 Department of Orthopedic Surgery, School of Medicine, Isfahan University of Medical Sciences, Isfahan, Iran

**Keywords:** Acute medial meniscus injury of the knee, Ultrasonography, Magnetic resonance imaging

## Abstract

**Background:**

In recent years, musculoskeletal ultrasound has increasingly become the common method for diagnosis for many medical specialties. Therefore, the present study was performed to evaluate the diagnostic value of point-of-care ultrasonography (POCUS) as a primary triage tool in the diagnosis of the acute medial meniscus injury of the knee.

**Materials and methods:**

The present cross-sectional study was performed on patients with a suspected medial meniscus injury of the knee in the emergency department (ED). After history taking and primary physical examination, radiographic imaging of the knee was done. If there was no fracture in the knee X-ray, the POCUS examination on the knee was carried out. All the patients were asked to refer to an orthopedic clinic 2 weeks after discharge from ED for the Magnetic Resonance Imaging (MRI) evaluation. Finally, the POCUS findings were compared with the MRI findings in diagnosing medial meniscus injury.

**Results:**

Fifty-five patients with a mean age of 35.48 ± 11.58 years were analyzed in the study (69.1% male). In comparison with MRI scan, the sensitivity and specificity of POCUS in the detection of medial meniscus injury were 85.0 [95% confidence interval (CI), 54.0 to 98.9] and 65.7% [95% CI 42.2 to 85.7], respectively. Its positive and negative predictive values were 58.6% [95% CI 33.8 to 81.5] and 88.5% [95% CI 62.1 to 99.3], respectively. (Area under the ROC curve = 0.726, *P* value = 0.003).

**Conclusion:**

The present study demonstrated that POCUS can reasonably be applied in comparison with MRI to evaluate medial meniscus injury. POCUS is an effective initial diagnostic modality in patients with suspected medial meniscus injuries.

**Supplementary Information:**

The online version contains supplementary material available at 10.1186/s13089-021-00256-0.

## Introduction

Knee injuries are a common presenting concern to the emergency department (ED) [1]. Meniscus injury, especially medial one, is one of the most common knee injuries. The meniscus is one of the most significant parts of the knee, plays the role of shock absorber in the knee, and strengthens the knee. Therefore, meniscus injury of the knee can interfere with the proper functioning of the knee and negatively affect patients’ daily life until their recovery. For instance, the United States and Great Britain have reported that meniscal injuries have an incidence rate of 61 cases per 100,000/year and 23 cases per 100,000/year, respectively [1, 2].

Magnetic resonance imaging (MRI) has historically been the gold-standard imaging tool for non-invasive detection of meniscus damage [3]. However, MRI is not only expensive and unavailable in many places, but also has significant limitations, such as metallic implants and cardiac pacemakers, claustrophobia, artifacts, a long examination time, and delay in treatment due to a long waiting period [1, 4, 5]. Arthroscopy, as the main diagnostic standard test, is used to establish meniscal injuries. Arthroscopy is associated with some limitations. Yet, it is invasive, costly, and needs hospitals [6, 7].

In addition, in recent years, ultrasonography (US) for evaluating patients with suspicious soft tissue or bone injuries has become increasingly recognized for its diagnostic value in the ED setting due to its accuracy [8]. The structures of the ankle are superficial and can be easily evaluated by the US [7, 8]. Point-of-care ultrasound (POCUS) is an alternative, inexpensive, non-invasive, easily available, and real-time imaging tool to identify the soft tissue pathology of the knee, including medial meniscus injuries [9, 10]. US examination has been performed for the diagnosis of meniscal injuries of the knee for over two decades [1, 10, 11]. US can be performed easily in the ED without time-consuming, especially in trauma patients to determine the treatment plan and whether surgical intervention [2].

Other advantages of the US examination include the lack of ionizing radiation, focused evaluation correlated with the specific site of pain and trauma, and utility in patients for whom MRI is contraindicated [1, 4, 5]. The US examination is a possible alternative to MRI, and it can be done faster and cheaper to evaluate meniscus. The use of POCUS for diagnosing meniscal tears has been proposed, but the diagnostic accuracy of ultrasound for knee meniscus injuries has remained controversial [1, 5, 8]. The sensitivity and specificity of US in the diagnosis of meniscus injuries have indicated a wide range in previous studies [12–15]. Moreover, some studies have reported the diagnostic value of US, as compared with MRI, in diagnosing meniscus injuries to be weak and inappropriate [12, 16, 17], and some have reported it to be very useful and appropriate [2, 15, 18, 19].

Given the fact that knee injury is the common injury in the ED and a controversial issue in previous studies, the present study aimed to compare the efficacy of POCUS, in detecting medial meniscus injury in the patients with acute knee trauma in the ED.

## Materials and methods

### Study design and setting

This prospective cross-sectional study was conducted between 2020 and 2021 at the emergency department (ED) of Kashani hospital in Isfahan, Iran. The study received ethics approval from the ethics committee of Isfahan University of Medical Sciences (IR.MUI.MED.REC.1399.879).

### Participants

All patients admitted to the ED with acute knee injuries and suspected acute MCL injury by historical and clinical examination when a trained emergency medicine specialist was available were eligible for enrollment into the study. Suspected acute medial meniscus injury has been defined as acute trauma, pain, tenderness, and swelling of the medial knee, and locked knee. A convenience sample of patients was enrolled from 8 AM to 8 PM every day within the week.

Patients older than 18 years and hemodynamically stable admitted with suspected acute medial meniscus injury following acute blunt knee trauma were included in this study. The patients with multiple trauma, loss of consciousness, history of a previous medial meniscus tear or previous injury on the injury site, diagnosis of a fracture in the knee, and those who declined to participate in the study and refused to continue treatment and orthopedic follow-up were excluded. In addition, the patients who were not alert, were not able to cooperate, and presented after 5 days of knee injury were not enrolled in the study.

### Study protocol

After obtaining written consent from eligible patients to enter the study, first, the demographic data (age and sex) were recorded. Then, routine physical examinations of the knee were performed. After history taking and primary physical examination, a data collection sheet that included physical examination findings was completed, and then two-view radiographic imaging of the knee (AP and lateral X-ray) was done. If there was no fracture in the knee X-ray, the point-of-care sonographic (POCUS) examination on the knee was carried out. Ultrasound was performed with Philips Affiniti 50G ultrasound machine with L12-5 Liner probe (5–12 MHz) by a trained emergency medicine specialist.

**Fig. 1:**
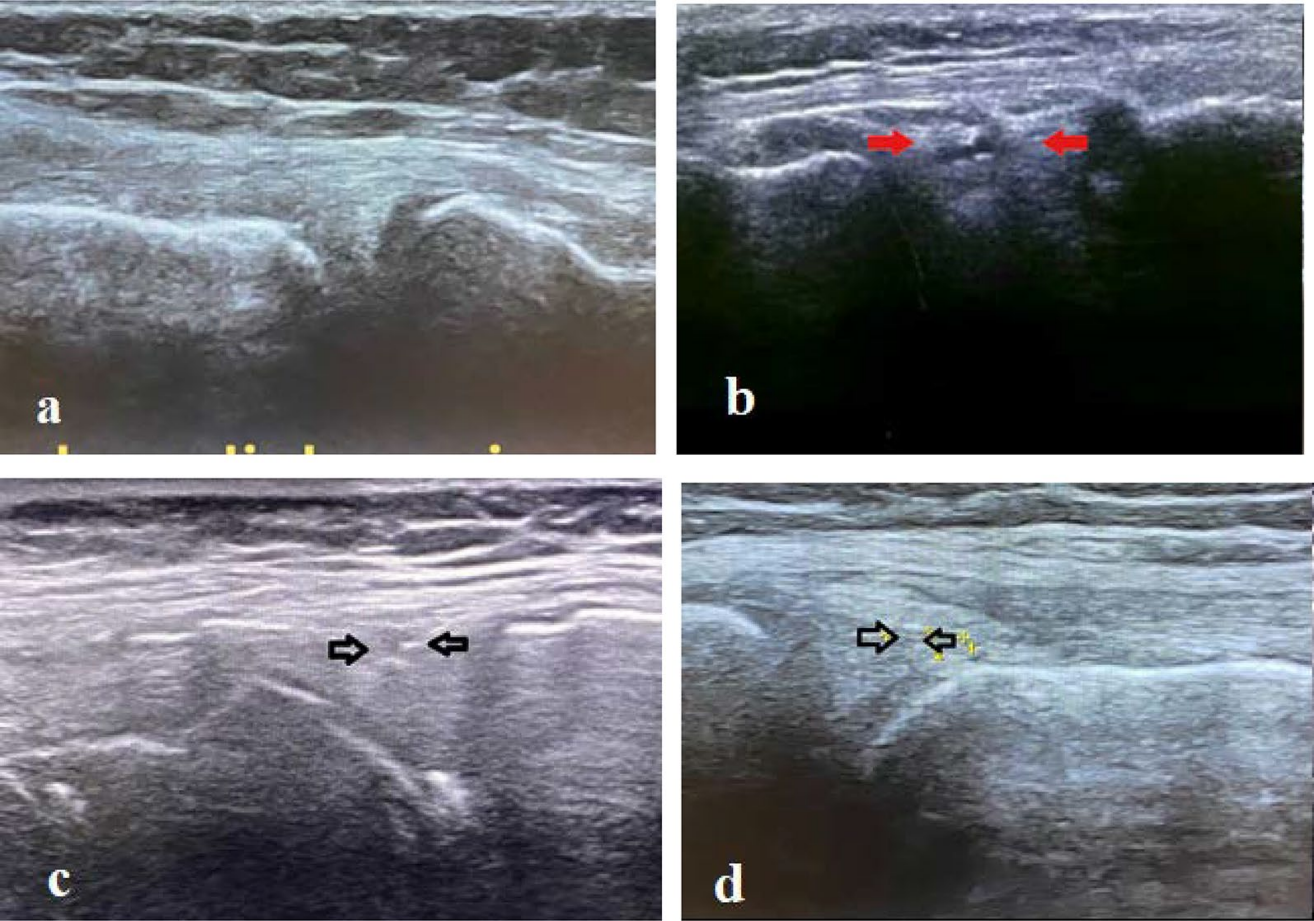
**a** Normal medial meniscus, **b**–**d** medial meniscus tears

The patients were supine, with the knee of interest in 45–90 degrees of flexion. Ultrasound images were done at the medial aspect of the knee using a longitudinal plane parallel to the medial collateral ligament. The medial meniscus is observed in the form of a wave between the two parts of the hyperechoic structures caused by the distal femur and proximal tibia. The normal medial meniscus is homogenous with no fluid around it. The tear of the medial meniscus is seen as a tear or cleft without echo or hypoechoic (Additional file 1). Therefore, the presence or absence of medial meniscus injury was recorded for each patient (Fig. 1).

After the POCUS examination, the ultrasonography findings were recorded on data collection forms. Treatment of knee injuries was always conducted with a cylindrical splint of the lower limb. All the patients were asked to refer to an orthopedic clinic 2 weeks after discharge from ED. The patients’ address and telephone number were recorded for the follow-up, and then the patient was discharged according to the standard of emergency care and the routine treatment plan of the emergency department, the patient referred to the orthopedic clinic with prior coordination and arrangement. If no referral was made, the patient was followed up by telephone and was encouraged to have an MRI evaluation at the clinic.

The MRI machine used in this study was GE Tesla (General Electric Company of America). The Quadknee coil specific for the knee was used, as well. In the orthopedic clinic, the patient was examined by a specific orthopedic specialist who was blinded to the POCUS results, and the diagnosis of medial meniscus tear was made based on MRI findings. The radiologists who were blind to the POCUS findings evaluated the MRI.

In addition, it should be noted that to eliminate the possible effect of the individual skill and device quality in recording the findings of medial meniscus injury, all ultrasounds were performed in a single center by a single emergency medicine specialist. Moreover, all the follow-up were performed in one center by a single orthopedic specialist. The collected variables, including age, sex, clinical findings, POCUS, and MRI findings, were recorded on the data collection forms. Finally, the POCUS findings were compared with the MRI findings in diagnosing medial meniscus injury.

### Sample size

The sample size of 45 patients was calculated at the confidence interval of 95%, test power of 80%, and it was based on the results of previous studies [14, 19] indicating the sensitivity of 0.7 and 0.9 and the error level of 0.2. Thus, the study population of 60 patients was selected for an anticipated dropout rate of 20% to ensure an adequately powered study. All patients who met the inclusion criteria were enrolled in the study until reaching the calculated sample size.

### Statistical analysis

Finally, the collected data were entered into SPSS software (Ver. 25) and was presented as n (%) or means ± standard deviation (SD). ROC analysis was used to evaluate the diagnostic value of sonography, clinical findings, and the combination of these two criteria. The area under the curve (AUC) indices, sensitivity and specificity, positive and negative predictive values (PPV, NPV), and positive and negative likelihood ratios (+ LR, − LR) were extracted. The significance level of less than 0.05 was considered in all analyses.

## Results

**Fig. 2:**
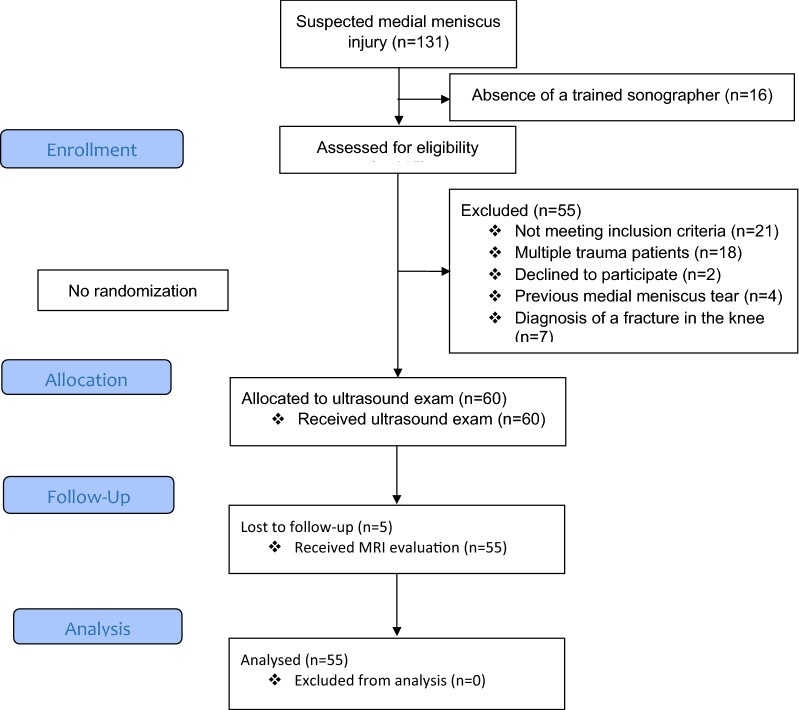
CONSORT flow diagram

A total of 131 patients with a suspected medial meniscus injury after acute blunt knee trauma were admitted to the ED. Among them, 16 were not eligible for the study due to the absence of a trained sonographer, and 55 were excluded. Finally, 60 patients were enrolled in the study. Five POCUS-negative patients did not have any orthopedic follow-up due to relative improvement in symptoms and the presence of the Covid-19 pandemic. Therefore, these patients did not undergo MRI and the final analysis was performed on 55 patients. The study flow diagram is shown in Fig. 2.

The patients were 38 (69.1%) male and 17 (30.9%) female with a mean age of 35.48 ± 11.58 years. The patient’s demographic and clinical findings were presented in Table 1. According to the MRI findings as to the gold standard, 20 (36.4%) patients had medial meniscus injury while the POCUS showed 29 (52.7%) patients had medial meniscus injuries (Table 1).

**Table 1:** Basic characteristics of the patients in this study

Variable	
Sex, No. (%)	
Male	38 (69.1%)
Female	17 (30.9%)
Age; year	35.48 ± 11.58
Clinical findings of knee, no. (%)	
Pain	55 (100%)
Tenderness	55 (100%)
Swelling	55 (100%)
Medial meniscus tears found on ultrasonography, no. (%)	
Yes	29 (52.7%)
No	26 (47.3%)
Medial meniscus tears found on MRI, no. (%)	
Yes	20 (36.4%)
No	35 (63.6%)

**Fig. 3:**
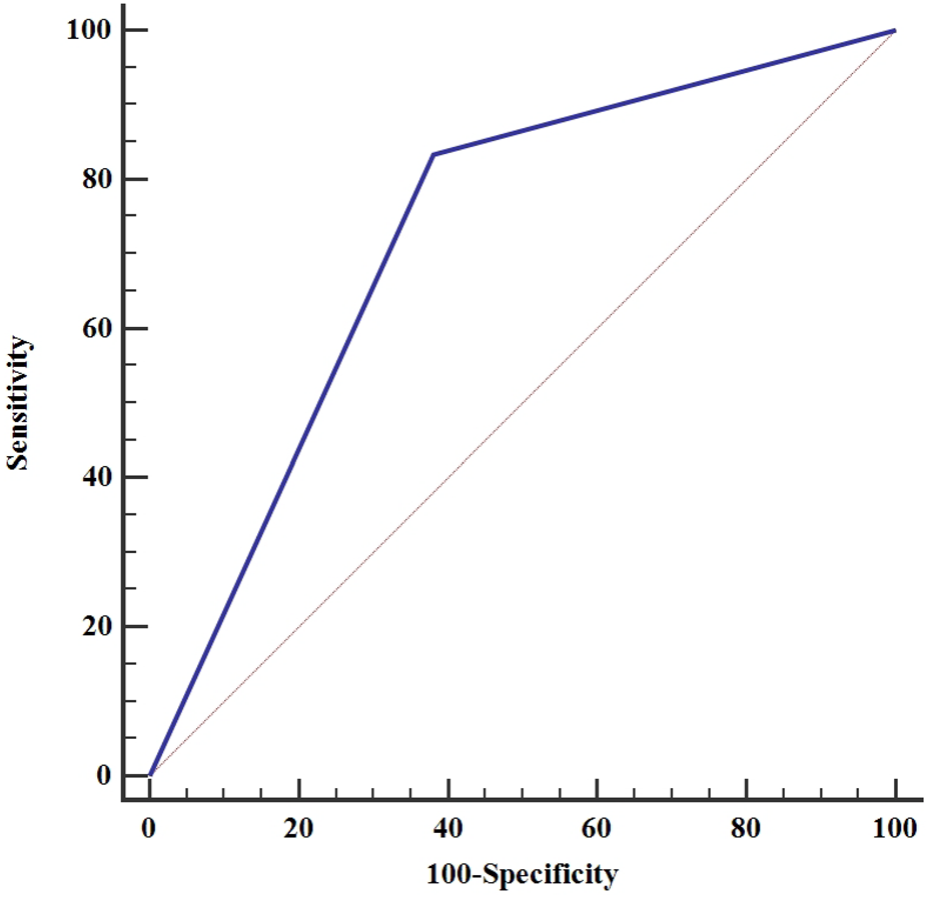
Rock curve in the diagnosis of medial meniscus injury with ultrasound criteria. A convenience sample of patients with elbow injuries necessitating radiographic evaluation for fracture, presenting when a trained study physician was available, was eligible for enrollment. Our study has some limitations. First, we included a convenience sample of patients enrolled when a trained enrolling physician was available; nonetheless, we believe that our sample is a generalizable group of patients, given that our findings are comparable with previously published data

In comparison with MRI scans, the sensitivity and specificity of ultrasound in the detection of medial meniscus injury were 85.0 [95% confidence interval (CI), 54.0 to 98.9] and 65.7% [95% CI 42.2 to 85.7], respectively. Its positive and negative predictive values (PPV and NPV) were 58.6% [95% CI 33.8 to 81.5] and 88.5% [95% CI 62.1 to 99.3], respectively. The area under the receiver-operating characteristic (ROC) curve of the POCUS exam in the detection of medial meniscus injuries was 72.6, *P* value = 0.003 (Table 2 and Fig. 3).

**Table 2:** Diagnostic value of ultrasound in the diagnosis of medial meniscus injury

MRI findings	Ultrasonography findings	
	Positive (*n* = 29)	Negative (*n* = 26)
Positive (*n* = 20)	17	3
Negative (*n* = 35)	12	23

Parameters of ROC analysis	Ultrasonography
AUC [95% CI]	0.726 [0.573 to 0.879]
*P* value	0.003
Sensitivity, % [95% CI]	85.0 [54.0 to 98.9]
Specificity, % [95% CI]	65.7 [42.2 to 85.7]
PPV, % [95% CI]	58.6 [33.8 to 81.5]
NPV, % [95% CI]	88.5 [62.1 to 99.3]
-LR, % [95% CI]	0.27 [0.07 to 1.0]
+ LR, % [95% CI]	2.19 [1.2 to 4.0]

*AUC* Area under curve, *PPV* Positive predictive value, *NPV* Negative predictive value, −*LP* Negative Likelihood ratios, + *LR* Positive Likelihood ratios, *CI* confidence interval

## Discussion

In recent years, musculoskeletal US has increasingly become the method of choice for diagnosis in many medical specialties such as sports medicine, rheumatology, anesthesia, and pain medicine. Various studies have shown the efficacy of US in the diagnosis of different ligaments injuries. In addition, the use of POCUS as the primary triage tool for the diagnosis of knee injuries has several benefits, such as being non-invasive, cost-efficient, faster to obtain than an MRI study, in addition to its procedure that allows for real-time diagnosis and early management of injuries [2, 3].

The results of the present study revealed that, in general, 36.4% of patients with suspected acute medial meniscus injury of the knee had meniscus injury (confirmed by MRI as the gold-standard method). The medial meniscus tears were detected among 15.3% to 88% of patients with medial knee trauma and pain [18]. In the present study, the sensitivity, specificity, PPV, and NPV of POCUS in diagnosing this injury were 85.0%, 65.7%, 58.6%, and 88.5%, respectively.

Previous studies demonstrated that overall US examination sensitivity and specificity for medial meniscus tears ranged from 83 to 97.2%, and 83 to 100%, respectively [13, 15, 19]. Alizadeh et al. prospectively evaluated 74 consecutive patients with clinical suspicion of medial meniscal tear, in two different groups. They showed that the sensitivity, specificity, PPV, NPV, and accuracy of US in detecting medial meniscal tears in patients ≤ 30 years were 100%, 88.9%, 96.5%, 100%, 97.3% and in patients > 30 years were 83.3%, 71.4%, 92.6%, 50%, 81.1%, respectively [1]. Therefore, they recommended the US examination/technique as an effective initial investigation for medial meniscus injuries in younger patients (≤ 30 years). Most of the patients in the present study were over 30 years old, which may be due to the low specificity of ultrasound. In older patients, mucoid degeneration in the medial meniscus, decrease in cartilage thickness, and marginal osteophytes around the knee may limit the ultrasound view.

Similar to the current study, Ghosh et al. evaluated 9 patients with a median age of 53 years by POCUS before the MRI. POCUS showed 100% sensitivity and 50% specificity for medial meniscus tear [19]. In another study, the sensitivity, specificity, PPV, and NPV of sonography in comparison with arthrography for diagnosing medial meniscus tears were 75%, 88%, 80%, and 85% [20]. Our results find support from these studies.

Omer et al. demonstrated that sensitivity, specificity, PPV, NPV, and diagnostic accuracy of US for medial meniscus tears were 95.00%, 73.68%, 79.16%, 93.33%, and 84.61% [18].

Inconsistent with the present study, several studies demonstrated that US exams of the knee in general radiological practice do not offer significant information above clinical examination [12, 17]. These studies were done in 2002 and 2004; however, the studies performed more recently have reported higher sensitivities and specificities of US in diagnosing medial meniscus tears, which may be attributed to technology and increased operator training.

However, according to many experts’ opinions, most meniscus injuries can be visualized through ultrasound if the patient is positioned correctly and the appropriate transducer is selected. In this way, the sensitivity of ultrasound in the diagnosis of medial meniscus injury in the present study was 85.0%.

In meta-analysis by Xiao et al. which included 21 studies, sensitivity, specificity, and area under curve (AUC) of ultrasonography diagnosis were 0.775 (95% CI 0.747–0.801), 0.838 (95% CI 0.818–0.857), and 0.9107 (95% CI 0.8625–0.9589), respectively. They suggested ultrasonography should be routinely used for the evaluation of medial meniscal injuries in the knee joint [11]. Dai et al. who conducted a meta-analysis including 7 prospective studies (*n* = 551), demonstrated that the ultrasonography in the diagnosis of meniscal injury had sensitivity, specificity, positive likelihood ratio, and negative likelihood ratio of 0.88 (95% CI 0.84–0.91), 0.90 (95% CI 0.86–0.93), 7.07 (95% CI 4.34–11.52), and 0.17 (95% CI 0.10–0.26), respectively [21]. In contrast to the present study, these meta-analyses had high specificity but moderate sensitivity. The slightly low specificity of POCUS in the present study (65.7%) may indicate low sample size and poor technique and may improve with increasing experience with the method. The specificity of ultrasound was lower in the first stage of the study and as experience increased, the false-negative also decreased. Based on the results of the present study, POCUS may have a role as the initial modality in patients with suspected medial meniscus tears because of sensitivity of 85.0%, and it may serve as an effective screening tool for patients with acute knee trauma.

In the current study, NPV for using ultrasound to diagnose medial meniscal tears was 88.5% which was lower than previous studies [15, 19, 22]. Alizadeh et al. showed the NPV of 100% and 50% in patients ≤ 30 years and > 30 years [1]. In addition, Shetty et al. found an NPV of 75% for the US in the diagnosis of meniscal tears [23]. The difference in NPV of US in detecting medial meniscus tears between studies could be related to several factors, such as differences in age of study population, sonographer experience, probe and ultrasound machine, and the time of knee trauma (acute or chronic).

Finally, we recommend using POCUS as a screening tool and first-line modality for patients with acute knee trauma and clinical suspicion of medial meniscus injuries. In the patients with negative results, if there is no improvement after 2 weeks, the next step is MRI examination to rule out medial meniscus injuries because of NPV of 88.5% in POCUS. In the patients with positive results, it is recommended to further refer to MRI because of the high probability of medial meniscus tears. POCUS is a widely available and accurate and reliable diagnostic tool, therefore, POCUS is considered a good low-cost alternative when MRI is a contraindication or not available or when waiting time for MRI can cause unnecessary delay in management.

In the present study, emergency medicine specialists were trained to perform POCUS in diagnosing this injury. In addition, all POCUS were performed by a single device with high accuracy. Therefore, operator skills, as well as the device, did not have a distorting effect in the present study, which can be regarded as one of the strengths of this study.

### Limitations

The main limitation of the present study was the small number of subjects that were enrolled. The sample size was calculated based on the results of the previous studies and due to the influence of sample size on diagnostic accuracy, it is thus recommended that the study be performed with larger sample size and multicenter. In addition, the only medial meniscus was evaluated. It was better to evaluate the medial compartment of the knee with ultrasound to determine the simultaneous tear of the medial collateral ligament. Finally, in this study, MRI was considered the gold standard and patients did not undergo arthroscopy. The accuracy of MRI in the diagnosis of meniscal tears was dependent on the experience of the interpreter. Arthroscopy was not performed routinely at our institution and not all patients needed arthroscopy. Of course, the accuracy of MRI, compared with arthroscopy, is 97%, but the comparison of ultrasound and arthroscopy has more accurate results.

## Conclusion

The present study demonstrated that POCUS is a useful adjuvant diagnostic modality to evaluate medial meniscus injury POCUS helps in taking a decision regarding management of a medial meniscus tear as the patient can avoid performing the costly and time-consuming confirmatory MRI if the result of POCUS is negative. In the patients with negative results, the next step is an MRI examination if there is no improvement after 2 weeks. Therefore, POCUS is recommended as an effective initial investigation in the patients suspected of having medial meniscus injuries.

A further large-scale study is suggested by improving the technique to establish the diagnostic accuracy of US in detecting meniscal injuries.

## Supplementary Information

**Additional file 1.** Medial meniscus point-of-care ultrasonography.

## Data Availability

Yes.
